# Medical Error Reporting among healthcare workers in a Kenyan tertiary level hospital: a knowledge, attitude, and practice study

**DOI:** 10.1186/s12913-025-13886-0

**Published:** 2025-12-17

**Authors:** Lydia Okutoyi, Pamela Godia, Mary Adam, Fred Sitati, Walter Jaoko

**Affiliations:** 1 University of Nairobi, Nairobi, Kenya; 2 Healthcare Quality Division, Kenyatta National Hospital, Nairobi, Kenya; 3 Kijabe Mission Hospital, Nairobi, Kenya; 4 ACQUIRE, The African Consortium for Quality Improvement Research in Frontline Healthcare , Nairobi, Kenya

**Keywords:** Patient-safety, Medical-error, Reporting, Practice, Attitude

## Abstract

**Background:**

Medical Error Reporting (MER) enables organizations to characterize safety events, learn from them, and mitigate their recurrence in the future. However, Medical Error Reporting is inconsistently practiced by healthcare workers. This study aimed to assess the knowledge, practice, and attitude towards MER at the Kenyatta National Hospital (KNH), a tertiary care teaching and referral hospital in Kenya that serves Kenya and the East and Central African regions.

**Methods:**

This cross-sectional study was conducted among healthcare workers at KNH between February and July 2022. Out of a calculated sample size of 384, a total of 390 participants were recruited, and 372 were included in the final analysis. Stratified convenience sampling was used to ensure representation across clinical cadres (nurses, doctors, and others) and hospital divisions (medicine, surgery, pharmacy, and others). Participants were recruited via email, departmental WhatsApp groups, or during meetings. Data were collected using a pre-tested, self-administered questionnaire (online and paper-based).

**Results:**

Of the 372 participants, most were nurses 62.3%, followed by doctors (30.3%) and other staff (7.4%). Awareness of the MER tool was reported by 259 (71.2%). A total of 247 (68.6%) had encountered a medical error in the past two years, and among them, 138 (55.9%) had used the MER form, submitting a total of 758 reports. Nearly half of the respondents (49.2%) expressed a positive attitude toward medical error reporting (MER), which was significantly associated with reporting behaviour, particularly among respondents in the Surgery division (χ² = 11.78, *p* = 0.003). Most participants, 245 (74.2%), correctly defined patient safety. MER use was significantly associated with cadre; nurses reported more than doctors (χ² = 25.1, df = 2, *p* < 0.001).

**Conclusion:**

Medical error reporting remains underutilized at KNH, especially among doctors. As the first study to document MER form use at the institution, it highlights gaps in awareness and attitude. Enhancing uptake will require addressing fear of victimization, strengthening supportive reporting cultures, and raising awareness across all healthcare worker cadres.

**Supplementary Information:**

The online version contains supplementary material available at 10.1186/s12913-025-13886-0.

## Introduction

Patient safety has received much attention in the past two decades. It has been over 20 years since the Institute of Medicine (IOM) publication *To Err is Human*,* Building Safer Health Systems.* This report exposed the scope of medical errors and their impact on patients, documenting that such errors are common, costly, and preventable [1]. While medical errors in and of themselves may or may not cause harm (near-miss events), a substantial number of errors do cause harm, resulting in disability or death [2].

Globally, about 1 in 10 persons experience an adverse event during care, causing an estimated 3 million deaths yearly [3, 4]. Harm during care in low- and middle-income countries (LMIC) had been underestimated; however, a multi-site study in 28 hospitals across eight countries in 2011 shed light on this topic [5]. In this study, 2.5% to 18.4% of the files reviewed per country recorded an adverse event, while one-third of these events reported an outcome of death [5]. A more recent publication showed, 134 million adverse events occur in low- and middle-income countries [3].

Reporting safety events has been utilized in the aviation industry for risk management for over four decades [6]. This approach was adopted later in health care; reporting adverse events is termed medical error reporting (MER) [6]. The primary purpose of error reporting in healthcare is to learn from errors and address system issues that might allow harm to happen. Much has been published on medical error reporting in high-income countries, but little is known about medical error reporting in LMIC [7]. In 2016, the World Health Organization established the minimum information reporting to guide reporting on medical errors [8]. However, the use of structured MER is relatively uncommon in sub-Saharan Africa SSA [2, 9]. Reporting of medication errors caused by adverse drug events in Africa has seen an early integration into structured reporting compared to the general medical errors [10]. When healthcare workers adopt the use of MER in a structured manner, more reports are submitted, thus enhancing learning, estimating the incidence, and enabling improvement [1].

At Kenyatta National Hospital (KNH), structured medical error reporting was introduced in 2017 through a paper-based, one-page Medical Error Reporting (MER) form. However, uptake among healthcare workers was initially low, mainly due to a lack of awareness, limited interest, and the perception that reporting was time-consuming or unnecessary. Previously, structured forms were used only for mandatory reporting of maternal mortality, adverse drug reactions (ADR), and adverse events following immunizations (AEFI), as required by the Ministry of Health (MOH), Kenya [11, 12]. Limited use of the MER form hampers system learning and patient safety. This study aimed to assess knowledge, attitudes, and practices toward MER at KNH and identify barriers to improvement.

## Methods

### Study design and setting

This was a cross-sectional survey among healthcare workers at Kenyatta National Hospital (KNH), the largest tertiary teaching and referral hospital in Kenya, serving the country and the wider East and Central African regions. The hospital has a capacity of about 2,000 beds and employs over 6,000 staff. It offers emergency and specialized care through 52 departments. Since 2017, a dedicated patient safety unit at KNH has supported the use of a structured Medical Error Reporting (MER) form. A work instruction on MER was developed to guide anonymous, non-punitive reporting, with a disclaimer assuring clinicians that information provided will not be used against them. The form excludes identifying details and is available in paper format in clinical areas for doctors, nurses, and other staff. Although an online MER system had been developed, it was not yet implemented during the study.

This study aimed to assess healthcare workers’ self-reported practices, knowledge, and attitudes toward the use of MER forms. It did not involve the analysis of submitted MER forms or validation of self-reported practices. A two-year recall period was considered reasonable for near-accurate responses. The survey was conducted over six months, from February to July 2022.

### Participants and sampling

The study population included doctors, nurses, pharmacists, technologists, and clinical officers involved in patient care at Kenyatta National Hospital (KNH), regardless of their affiliation with the University of Nairobi or the hospital side of the institution. The target sample size of 384 participants was derived using Cochran’s formula for prevalence studies, assuming a 30% estimated prevalence, 95% confidence level, and 5% precision. Stratification was based on cadre and division: medical nurses (120), surgical nurses (120), medical doctors (50), and surgical doctors (50), and (44) from other support disciplines to reflect the distribution of staff across the two major clinical divisions.

Within each stratum, convenience sampling was used. Participants were recruited through departmental meetings, WhatsApp groups, email, and in-person outreach by trained research assistants. Both electronic and paper-based questionnaires were used to accommodate varying preferences; online links were shared via departmental WhatsApp groups and email, while physical copies were distributed directly in clinical areas. Doctors generally preferred the online version, while nurses favored paper-based forms, citing inconsistent internet access in some hospital sections. Research assistants were assigned specific departments to minimize overlap and avoid duplicate responses. Online forms required email addresses to detect repeated entries, while paper questionnaires were uniquely coded.

Written informed consent was obtained from all participants—physically for paper forms and digitally through a consent prompt embedded in the online survey. This blended recruitment strategy and multimodal approach ensured a wide reach across cadres and departments, while respecting staff availability, preferences, and working conditions during data collection.

### Data collection

Data was collected over a 6-month period using a self-administered questionnaire that primarily captured quantitative data, with a few sections allowing for brief narrative responses within defined frameworks. The questionnaire was developed using knowledge from the literature on factors that could affect medical error reporting. It was pretested on 11 clinical healthcare workers, representing the two main clinical divisions, Surgery and Medicine, and the two main disciplines, nursing and doctors. Pilot data were not included in the final analysis. This questionnaire has not been published elsewhere; this is the first time it has been published. Participants could choose either a paper or an online version of the questionnaire. Written informed consent was obtained from all participants prior to their participation and was captured in both online and paper formats.

The main variables of interest included: use of the MER form, knowledge of who should fill out the form, correctness in the definition of patient safety, and attitude towards MER in the respective departments. The other variables included: cadre, division, number of years in KNH, and hours worked in a week.

The factors that affected the practice of MER were assessed by (1) the number of encounters with MERs in practice, and (2) ever using a non-structured form. Near-miss reporting was captured using a 5-item Likert scale (Never, Rarely, Sometimes, Most of the Time, and Always). The 5-item response format was collapsed to a dichotomous variable for analysis. A positive response to reporting near-miss events was indicated by answering, “always” or “most of the time,” while a negative response was shown by “sometimes,” “rarely,” or “never.” Two types of near-miss events were assessed: (a) an event caught and corrected before reaching the patient, and (b) a medical error that reached the patient but caused no harm.

Knowledge of the definition of patient safety was measured using a short-answer format. The responses were graded as correct if they included at least two of these themes: (a) harm or risk or side effects, (b) reduction or minimization, (c) in healthcare, or patients or during care, (d) discipline, or efforts or measures to harm. Vague responses could include any of the 6 Institute of Medicine (IOM) quality dimensions: effective, efficient, timely, patient-centred, equitable, and well-coordinated care [3]. Any response that fell outside the scope of quality dimensions was considered incorrect.

Knowledge of who should report medical errors was assessed, and responses were reported by staff close to the event or by leadership. Staff close to the event was the correct answer.

Participants rated the attitude toward MER in the department, and responses were positive/negative/don’t know. Participants also provided open-ended answers to state why they rated the attitude of MER as positive or negative.

### Data analysis

Data analysis was performed using Jamovi 2.3.21 and R Studio 4.2.2, both open-source software. Continuous variables were summarized as median and range due to skewed distribution, and categorical variables as absolute numbers and percentages. Chi-square tests were used for inter-group comparisons of categorical variables. Associations between clinical cadre, prior experience with non-structured reporting, and awareness of the Medical Error Reporting (MER) system with ever-using the MER tool were examined using the Chi-square test to assess the strength of these associations.

### Ethical consideration

#### Ethics approval

was granted by the KNH-UON Ethics Review Committee (P847/10/2021), and a research permit was obtained from the National Commission for Science Technology and Innovation (Ref 517313). Informed consent was obtained from all voluntary participants. No human samples or experiments were involved.

## Results

A total of 390 questionnaires were submitted, with 177 (45.4%) in electronic format and 213 (54.6%) on paper. After excluding four duplicate and four incomplete electronic entries, 169 electronic submissions were retained. Additionally, ten submissions from students were excluded, leaving 372 questionnaires for final analysis (169 electronic and 203 paper-based).

The response rate for the paper questionnaires was 76%, while it was not possible to calculate a response rate for the electronic format, as it was shared via work-related WhatsApp platforms with an unknown total reach.

### Characteristics of the participants

Among the 232 nurses, 152 (65.5%) were nursing officers, the entry-level cadre and the most common nursing category at KNH. Nurse specialists accounted for 49 (21.1%), while nurse managers comprised 31 (13.4%). Of the 113 doctors, postgraduate registrars formed the largest group at 43 (38.1%), followed by specialists 39 (34.5%) and medical officers 31 (27.4%).

Participants had a median duration of 6 years at KNH (interquartile range, IQR: 3–20) and 4 years at their current workstation (IQR: 2–8). By departmental distribution, most respondents were based in the surgical division 191 (51.0%), followed by the medical division 130 (35.0%). Pharmacy and diagnostic services accounted for 14.0% collectively.

Regarding working hours, 194 (52.8%) worked more than 40 h per week, while 166 (42.2%) worked 30–40 h, and only 7 (1.9%) reported working less than 30 h weekly. (See Table 1)

**Table 1:** Participants characteristics

Division		*n* (%)	
	Surgery	191(51.0%)	
	Medicine	130(35.0%)	
	Others	36(10%)	
	Pharmacy	15(4.0%)	
Cadre			
	Nurse	232(62.3%)	
	Doctor	113(30.3%)	
	Others	27(7.4%)	
Doctors category			
	Specialist	39(34.5%)	
	Post-graduate doctors	43(38.1%)	
	Medical officer	31(27.4%%)	
Nursing categories			
	Nurse manager	31(13.4%)	
	Nurse specialist	49(21.1%)	
	Nursing officer	152(65.5%)	
Hours of work/week			
	< 30	7(1.9%)	
	30–40	166(42.2%)	
	> 40	194(52.8%)	
Years in KNH, Median (IQR*)	6(3–20)		
Year in station, Median (IQR*)	4(2–8)		

*IQR -Interquartile- Range

### The practice of medical error reporting

Among the 372 participants, 247 (68.6%) reported encountering a medical error within the past two years. Doctors had the highest proportion of reporting such experiences at 90 (80.4%), followed by nurses at 140 (63.1%).

Among the 247 participants who encountered a medical error in the past two years, 212(85.8%) reported the incident through non-structured means (e.g., verbal or informal documentation), while only 138(55.9%) used the formal MER tool. Nurses had the highest unstructured reporting rate at 138(98.6%), followed by other staff at 16(94.1%), and doctors at 58(64.4%). In contrast, use of the MER form was notably lower among doctors 22(24.4%) compared to nurses 102(72.9%). A total of 758 MER forms had been submitted by participants within two years. Nurses contributed 473 (62.4%), doctors 132 (17.4%), and other staff 153 (20.2%). See Table 2.

### Reporting of near misses

Only 80 (22.1%) of participants reported they would consistently document an error caught before reaching the patient. Similarly, 79 (21.8%) indicated they would report an event that reached the patient but caused no harm, indicating low willingness to report near-miss events. See Table 2.

### The knowledge of Medical Error Reporting

Most participants, 259(71.2%), were aware of the MER system at KNH. Awareness was highest among nurses 181(80.1%) and lowest among doctors 53(47.3%). When asked who should fill an MER form, 343(92.2%) correctly identified staff closest to the event (including those who noticed or were involved in the incident). Only 29(7.8%) attributed this responsibility to leadership or patient safety unit staff.

On defining patient safety, 245(74.2%) gave correct responses. Incorrect answers were more frequent among nurses 33(16.2%) compared to doctors 8(7.8%). See Table 2.

### Attitude to MER

A positive attitude toward MER was reported by 183 (49.2%) participants, highest among nurses 123(51.3%), while doctors were lower at 41(35.7%). A significant association was observed between positive attitudes and actual reporting (χ² = 11.778, *p* = 0.003). This association was most notable within the Surgical division (χ² = 6.434, *p* = 0.040). No significant link was found in other divisions.

**Table 2:** Practice (Last 2 years), knowledge and attitude to MER

	All *n* (%)	Nurse *n* (%)	Doctors *n* (%)	Others *n* (%)
**Practice**				
**Encountered a Medical error event**				
YES	247 (68.6)	140 (63.1)	90 (80.4)	17 (65.4)
NO	113 (31.3)	82 (36.9)	22 (19.6)	9 (34.6)
**TOTAL**	**360 (100)**	**222(100)**	**112 (100)**	26 (100)
**Any reporting (non-structured)**				
YES*	212 (**85.8**)	138 (**98.6**)	58 (**64.4**)	16 (**94.1**)
NO	35 (14.2)	2 (1.4)	32 (35.6)	1(5.9))
**TOTAL**	**247 (100)**	140 **(100)**	90 **(100)**	17 (100)
**Ever reported using MER tool**				
YES*	138 (**55.9**)	102 (**72.9**)	22 (**24.4**)	15 (**88.2**)
NO	109 (44.1)	38 (27.1)	68 (75.6)	2 (11.8)
**TOTAL**	**247 (100)**	140 **(100)**	90 **(100)**	17 (100)
Number of MER Submitted*	758 (100)	473 (62.4)	132 (17.4)	153 (20.2)
**MER (Near-Miss) reporting rate**				
**Reporting of Near Miss**,** Positive (Always and most of the time)**				
When a mistake is caught and corrected before reaching the patient	80 (22.1)	60(25)	12(10)	8(33)
When a mistake reaches the patient but does not cause harm	79 (21.8)	64(26)	11(9)	4(11)
**Knowledge**				
**Aware of the existence of the MER tool**				
YES	259 (71.2)	181 (80.1)	53 (47.3)	25 (96.2)
NO	105 (28.8)	45 (19.9)	59 (52.7)	1 (3.8)
**TOTAL**	**364 (100)**	**226 (100)**	**112 (100)**	26 (100)
**Who should fill out a MER form?**				
Any staff who first notices the event	343 (92.2)	218 (94.4)	103 (89.6)	23 (85.2)
Team leadership	29 (7.8)	13 (5.6)	12 (10.4)	4 (14.8)
**TOTAL**	**372 (100)**	**231 (100)**	**115 (100)**	**27 (100)**
**Definition of patient safety**				
Correct	245(74.2)	147(72.4)	81(78.6)	17(70.8)
Vague	40(12.1)	23(11.3)	14(13.6)	3(12.5)
Wrong	45(13.6)	33(16.2)	8(7.8)	4(16.7)
**TOTAL**	**330(100)**	**203(100)**	**103(100)**	**24(100)**
Attitude				
Attitude to MER in the wards				
Positive	183 (49.2)	123 (51.3)	41 (35.7)	19 (70.4)
Negative	130 (34.9)	85 (35.4)	40 (34.9)	5 (18.5)
Not Known	59 (15.9	24 (10.3)	32 (27.8)	3 (11.1)
TOTAL	**372 (100)**	**240 (100)**	**115 (100)**	27 (100)

MER Reporting Rate (%)* (MER users ÷ encountered errors) = **85.8%**

**Any reporting (non-structured) Rate**(%)*(Non structured reporting *÷encountered errors* ) *=* **55.9%**

### Reasons for a positive or negative attitude toward MER in their department

Out of 382 participants, 298 (78.0%) provided reasons for their department’s attitude toward MER. Of these, 153 (51.3%) cited reasons for a **positive attitude**. The most common was a *positive team culture that encourages learning from mistakes* (31.2%), followed by integration of MER into *performance appraisal* (10.6%). Other positive reasons included *awareness* (5.1%), a *non-punitive environment* (3.1%), and receiving *action and feedback* (1.7%).

Among those citing **negative attitudes** (140 participants, 47.8%), the most frequent reason was *fear of victimization* (24.7%). Other contributors included *lack of awareness* (10.3%), a *blame and shame culture* (6.2%), *negative departmental culture* (4.8%), and *fear of litigation* (2.0%). See Table 3.

**Table 3:** Reasons for attitude towards medical error reporting

*N* = 293	Reason	*n*(%)
Positive Attitude	Positive culture*	93(31.2%)
	Part of appraisal*	31(10.6%)
	Awareness	15(5.1%)
	Non-Punitive culture	9(3.1%)
	Action and feedback	5(1.7%)
	**Total positive**	**153(51.3%)**
Negative attitude	Fear of Victimization	72(24.7%)
	Lack of awareness	30(10.3%)
	Blame and shame culture	18(6.2%)
	Negative culture*	14(4.8%)
	Fear of litigation	6(2.0%)
	**Total Negative**	**140(47.8%)**
Grandtotal		**293(100%)**
Positive culture*	Learn from mistakes, and this adds value to patient safety	
Negative culture*	Fear of reporting, no impact of reporting	
Part of appraisal*	MER reports required as individual and departmental documented evidence for appraisal	

### Factors associated with the use of the Medical Error Reporting form

Chi-square tests identified significant associations between the use of the Medical Error Reporting (MER) form and three key factors. First, clinical cadre was significantly associated with MER reporting (χ² = 24.4, df = 2, *p* < 0.001), with nurses and other clinical staff showing higher proportions of MER form use compared to doctors. Second, prior use of non-structured reporting methods was strongly linked to MER form use (χ² = 110, df = 1, *p* < 0.001), with those who had previously used any informal or unstructured reporting channels being more likely to submit a MER form. Lastly, awareness of the institutional MER system was significantly associated with reporting behaviour (χ² = 53.9, df = 1, *p* < 0.001), as staff who were aware of the MER system were more likely to report than those who were unaware. These findings indicate that professional cadre, awareness, and non-structured reporting mechanisms are key determinants of MER form utilisation. See Table 4.

**Table 4:** Factors associated with the use of the medical error reporting forms (*n* = 362–365)

Factor	Categories	Used MER Form (*n*)	Did Not Use MER Form (*n*)	χ²	df	*p*-value
Cadre	Doctors	22	90	24.4	2	< 0.001
	Nurses	102	124			
	Others	15	12			
Non-Structured reporting	Yes	129	85	110	1	< 0.001
	No	9	140			
Awareness	Yes	128	129	53.9	1	< 0.001
	No	9	96			

Note: The denominator varies due to missing responses across variables

Non-structured: Ever reported using any other means that is not MER form (structured)

Awareness: Awareness about the existence of a MER reporting form at KNH

## Discussion

Medical Error Reporting continues to be a challenge in health provision in Kenya and across the globe. This study found a mismatch between the high number of error encounters and low reporting rates at Kenyatta National Hospital. The paper-based MER system was underutilized, with doctors less likely than nurses to report using it. Prior use of non-structured reporting and awareness of the structured MER system were linked to higher reporting.

### Practice

In this study, 68.6% of HCWs reported encountering a medical error in the last two years. This rate is higher than that reported in a Ugandan study, where 53% and 58% of HCWs reported encountering diagnostic and medication errors, respectively [13]. The existence of a structured paper-based MER system at KNH may account for the higher recognition and documentation of errors in our setting. In contrast, a study conducted in an Accident and Emergency (A&E) department in South Africa found that 100% of HCWs reported encountering errors, which is significantly higher than our findings [14]. The South African context benefits from a national and regional patient safety incident reporting platform, backed by policy, which may enhance awareness and reporting of errors [15]. This suggests that the availability of national guidance and institutional systems plays a critical role in shaping HCWs’ recognition and reporting of errors. Literature suggests that most HCWs will encounter a harmful event at least once in their professional lives, making these systems vital for patientsafety [16].

Use of the MER form among those who encountered a medical error, 55.9%. This is notably higher than the 27.4% reporting rate to the national Adverse Drug Reaction (ADR) system found in a large multi-site study in Addis Ababa, Ethiopia [17]. Unlike our study, which directly quantified reported incidents, the ADR study used Likert scales to assess likelihood of reporting. Despite both relying on structured tools, lack of awareness and inaccessible reporting systems were major barriers in the Ethiopian study [17]. Another study in Ethiopia found only 12.4% of HCWs had ever submitted a clinical incident report [18]. While differences in study methodology may explain the variation in results, the lack of a structured reporting system at the hospital in Addis Ababa likely also contributed to lower reporting rates.

In our study, 72.9% of nurses who encountered a medical error reported it using the MER form. This rate is higher than the 36.7% reporting rate among ICU nurses in three tertiary hospitals in KwaZulu Natal, South Africa, as reported by Mjadu et al. in 2017 [19]. A follow-up study by Gqaleni et al. in 2020 showed a marked improvement, with 84% of ICU nurses reporting incidents, reflecting a strengthening patient safety culture in South Africa over time [19, 20]. In contrast, Kenya’s national patient safety policy was introduced in 2022, just after our study’s data collection concluded. However, at the institutional level, KNH had a medical error reporting (MER) work-instruction in place for six years, likely contributing to higher nurse reporting rates observed in this study [21, 22].

In our study, nurses were significantly more likely to report using the MER form compared to doctors. This trend mirrors findings from other studies. Zoghby et al. reported that nurses were 21 times more likely to report than doctors [14]. A study in a U.S. children’s hospital showed nurses had nearly three times higher odds of reporting than physicians (OR: 2.8; 95% CI: 1.3–6.0) [23]. At KNH, some nursing units incorporate error reporting into performance metrics, which may contribute to higher reporting rates among nurses. In contrast, doctors, although more likely to encounter medical errors, reported them less frequently. This highlights a critical need for targeted strategies to improve physicians’ engagement in structured error reporting systems like the MER.

### Near miss

In our study, near-miss reporting was low: 22.1% reported errors caught before reaching the patient, and 21.8% reported errors that reached the patient without causing harm. In comparison, Engeda et al. reported higher rates among Ethiopian nurses; 41% for intercepted errors and 37% for non-harmful errors that reached patients [24]. Reporting near misses is essential for learning from potential harm and improving safety systems [25]. The low reporting rates in our setting indicate missed opportunities for institutional learning and patient safety enhancement.

### Knowledge

In this study, 71.2% of respondents were aware of the MER system. This is lower than the 97% reported by Gqaleni et al. among ICU nurses across ten hospitals in KwaZulu Natal, South Africa, but comparable to the 63.4% found by Mjadu et al. [19, 20]. In our study, awareness was notably higher among nurses 80.1% compared to doctors 47.3%. This contrasts with Afolalu et al., who found greater awareness among doctors 40% than nurses 10% [26]. Awareness and prior experience with non-structured reporting significantly increased the likelihood of MER form use in our study.

Concerning who should fill the MER form, 92.2% of the participants at KNH correctly identified that the person closest to the event, not leadership, should complete the MER form. This contrasts with findings by Afolalu et al., where 57.6% of nurses and 64.5% of doctors were either unsure or incorrectly believed someone else was responsible for reporting [26]. These results suggest stronger clarity on reporting roles among KNH staff.

### Attitude

In our study, only 49.2% of participants perceived their ward’s attitude toward MER as positive, while 34.9% perceived it as negative, and 15.9% were unsure. This contrasts sharply with a Ugandan study where 97.7% of healthcare workers held a strongly positive individual attitude toward MER [27]. Unlike that study, ours assessed perceived departmental attitudes, capturing how reporting culture is experienced collectively. A similar Nigerian study found 55% of nurses and 53.3% of doctors viewed MER as ineffective [26]. At KNH, we found that positive attitudes were significantly associated with higher reporting in the surgical division—a relationship not explored in the other studies.

### Barriers to medical error reporting

In our study, 47.8% of participants expressed a negative attitude toward MER. The top reasons were fear of victimization 24.7%, lack of awareness (10.3%), and a blame-and-shame culture 6.2%. These findings mirror results from other studies: Poorolajal et al. found that 60% of respondents cited a lack of effective MER systems, 56% cited lack of peer support, and 51.8% cited the absence of reporting tools as key barriers [28]. Similarly, fear of victimization has been repeatedly highlighted in literature as a major deterrent to reporting [20, 24]. These results underscore the need to address both systemic and cultural barriers—particularly by promoting a non-punitive environment and increasing awareness of the MER system.

#### Study limitations

The principal investigator’s role in the KNH Safety Unit may have introduced response bias, though this was reduced by using independent data collectors. Self-reporting and social desirability bias are additional limitations. The study also did not collect demographic variables such as age and sex, limiting assessment of their potential confounding effect on reporting behaviour. Future studies incorporating these variables and analysing actual MER entries or online systems may provide deeper insights.

## Conclusion

This study provides the first institutional-level assessment of medical error reporting practices at Kenyatta National Hospital, offering critical insights into awareness, usage, and attitudes toward MER tools. Despite availability, underutilization persists, particularly among doctors, driven by fear of victimization and negative departmental cultures. Fostering a non-punitive environment, strengthening awareness, and embedding MER into routine quality improvement processes can enhance reporting. Further research—particularly on the content and outcomes of submitted MER reports—will be essential in shaping a stronger patient safety culture across the hospital.

Stratified convenience sampling was used to ensure representation across clinical cadres (nurses, doctors, and others) and hospital divisions (medicine, surgery, pharmacy, and others).

### Recommendations

- Enhance awareness and training on the use of the MER tool.
- Enhance clinical governance to reduce victimisation and foster a supportive, blame-free work environment.

## Supplementary Information

Below is the link to the electronic supplementary material.

Supplementary Material 1

Supplementary Material 2

## Data Availability

The data described in the manuscript, including all relevant raw data, are freely available to any scientist wishing to use them for non-commercial purposes with limits to what the participants consented to use in their informed consenting form. I have attached the data, and the r-scripts used to analyze the data.
