# Supplementary material for: Medical Error Reporting among healthcare workers in a Kenyan tertiary level hospital: a knowledge, attitude, and practice study
Source: BMC Health Serv Res. 2025 Dec 17;26:102. doi: 10.1186/s12913-025-13886-0 (PMC12822039; doi:10.1186/s12913-025-13886-0)
Supplement: Supplementary file 1 — Supplementary Material 1 [file 12913_2025_13886_MOESM1_ESM.pdf]

ASSESING THE PRACTICE, ENABLERS AND BARRIERS TO MEDICAL ERROR REPORTING IN KNH

Please complete the survey below.

Thank you!

CONSENT INFORMATION DOCUMENT

Title: The impact of QI initiative on medical error reporting at KNH

Introduction
Hospitals are a hazardous environment due to the complexity of the care processes. Medical errors often occur even in the most resourced facilities. Incident reporting, having gained traction in other fields Aviation, Nuclear energy and Petrochemical industries has been adapted into healthcare as medical error reporting. Medical error reporting enables hospitals to identify and learn from mistakes and improve patient care. Structured reporting of medical errors was first introduced in KNH 3 years ago, this has been stated in the hospital policy documents and cascaded to each clinical area to identify and report these events in the hospital's MERs. However, MER is largely voluntary and only a small fraction of never events are mandatory. The performance of the MERs has been low, while insufficient sensitization and training of the staff has been a challenge.

General Study Objective
The main objective of this study is to evaluate the impact of QI intervention on medical error reports rates and types.

Specific objectives
1. To determine the rates and types of reported medical error Pre-intervention
2. To assess the practice, barriers, enabler and improvement options for medical error reporting
3. To compare rates of medical error reporting pre and post-intervention, and between the Control and Intervention sites
Risks
The information that you will provide during this study will be kept in confidence and there will be any anticipated risks whatsoever.

Benefits
Participating in this study and answering the questions will help increase my understanding of medical errors, the various intervention measures, and how well we can ensure patient safety. This information will be beneficial to health professionals, the government, and other relevant stakeholders to formulate strategies for patient safety.

The Principal investigator is Lydia Okutoyi from Health care Quality KNH, with the support of the Patient Safety Unit at Kenyatta National Hospital. Principal investigators contacts are 0721814381

If you have any concerns about how this study is being conducted, you can get in touch with the secretary of Kenyatta National Hospital-University of Nairobi Research and ethics Committee through the following contacts: KNH/UoN - ERC P.O. Box 20723-00203 or Email: uonknh_erc@uonbi.ac.ke or Tel. 726300-9 Monday to Friday from 9.00am to 5.00 pm.

CONSENT FORM

Voluntarism
Your participation in this study is voluntary and you

◯ Yes
◯ No

have the right to refuse to participate or answer any questions that you feel uncomfortable with. If you change your mind about participating during the course of this study, you have the right to withdraw at any time.

Declaration of the respondent
I have understood the purpose of this study and therefore consent voluntarily to participate as a respondent.

Kindly Share your email (Optional)

____

A. BACKGROUND INFORMATION

1. Do you provide care to patients at KNH?

- ◯ Yes
- ◯ No

2. Which department do you belong/ serve

- ◯ Paediatrics
- ◯ Internal medicine
- ◯ Renal medicine
- ◯ Cardiology
- ◯ Specialized medicine
- ◯ Orthopaedic surgery
- ◯ General surgery
- ◯ Obstetrics and Gynecology
- ◯ Ophthalmology
- ◯ Ear Nose and Throat (ENT)
- ◯ Anaesthesia
- ◯ Laboratory medicine
- ◯ TSSU/CSSD
- ◯ Theatres
- ◯ Pharmacy
- ◯ Radiology
- ◯ Others
- ◯ KPCC

Any other department not listed specify

____

3. Are you, Doctor or Nurse

- ◯ Doctor
- ◯ Nurse
- ◯ Others

If you're a doctor

- ◯ Doctor specialist
- ◯ Resident Registrar
- ◯ Doctor Medical officer
- ◯ Doctor Medical officer Intern
- ◯ Medical student

If a Nurse

- ◯ Nurse manager
- ◯ Nurse specialist
- ◯ Nursing officer
- ◯ Student nurse for a speciality training
- ◯ Student nurse

If others specify profession

____

4. How many years have you worked/ given care at KNH (Specify, number) __________________________

5. What are the number of years in the current station/ unit (Specify, number) __________________________

6. Typically how many hours do you work per week?
- ◯ Less than 30 hours per week
- ◯ 30 to 40 hours per week
- ◯ More than 40 hours per week

B. GENERAL KNOWLEDGE IN PATIENT SAFETY

1. In your own words, define or describe is Patient Safety (Statement) __________________________________

2. Give at least 2 reasons why medical errors or harm occur to patients. __________________________________

How often do the following errors occur in your area of work

	Never	Rarely	Sometimes	Always	Most of the times	I don't know
1. Diagnostic errors (Not limited to laboratory or radiology but through out patient evaluation and care)	◯	◯	◯	◯	◯	◯
2. Medication errors (Prescribing, dispensing, administration, dosage, Stock out)	◯	◯	◯	◯	◯	◯
3. Unsafe surgical procedures	◯	◯	◯	◯	◯	◯
4.Hospital care associated Infection (Surgical site infection, Blood stream infection, Ventilator associated infection, Catheter associated infection)	◯	◯	◯	◯	◯	◯
5. Unsafe Injection practices	◯	◯	◯	◯	◯	◯
6. Unsafe transfusion practices	◯	◯	◯	◯	◯	◯
7. Radiation errors	◯	◯	◯	◯	◯	◯

C. ATTITUDE OF MEDICAL ERROR REPORTING

1. What is the existing attitude towards medical error reporting in your department/unit

◯ Positive
◯ Negative
◯ Don't know

2. Give at least 2 reasons for your answer

____

D. The Practice of medical error reporting in your department/ unit

	Never	Rarely	Sometimes	Always	Most of the times	I don't know
1. We are informed about errors that happen in this unit	◯	◯	◯	◯	◯	◯
2. When errors happen in this unit we discuss ways to prevent them from happening again	◯	◯	◯	◯	◯	◯
3. We are informed of changes that happened because of the reports	◯	◯	◯	◯	◯	◯
4. Staff speak up when they see something that can negatively affect the patient	◯	◯	◯	◯	◯	◯
5. Staff speak up when a senior is doing something unsafe	◯	◯	◯	◯	◯	◯
6. Those in authority listen when staff raise patient safety concerns	◯	◯	◯	◯	◯	◯
7. Staff are afraid to ask question when something does not go right	◯	◯	◯	◯	◯	◯

E. PRACTICE OF MEDICAL ERROR REPORTING AT AN INDIVIDUAL LEVEL

1. In the past 2 years, How often have you encountered/ witnessed a patient under your care who has had a safety (As an individual staff) (Specify number)

____

2. In the past 2 years, how often have you witnessed/encountered a patient who had a safety event in your ward? (At a ward or unit, or clinic level- whether through reports or discussed with ward colleagues) (Specify number)

____

If Yes How did you report	◯ 1 Reported verbally to my supervisor<br>◯ 2 Wrote a report or a statement on a paper<br>◯ 3 Documented in a patients notes<br>◯ 4 Filled the KNH Medical error form<br>◯ 1 and 2<br>◯ 1 and 3<br>◯ All the above
3. Have you ever reported any patient safety event in the past 2 years?	◯ YES<br>◯ NO
4. Are you aware of the existence of a Medical error reporting system (Paper-based) in KNH	◯ YES<br>◯ NO
5. Who do you think should fill the medical error forms (Choose the best option)	◯ Staff closest to the event<br>◯ Staff who was involved in inpatient care at the time of the event<br>◯ Any staff who notices that error has or had occurred<br>◯ The team leader of the shift<br>◯ Patient safety unit staff
6. How many times have you filled the MER in the last 2 years (Specify, number)	______________________

F. NEAR MISSES

	Never	Rarely	Sometimes	Always	Most of the times	I don't know
a. When a mistake is caught and corrected before reaching the patient how often is it reported?	◯	◯	◯	◯	◯	◯
b. when the mistake reaches the patient but does not cause harm, how often is it reported?	◯	◯	◯	◯	◯	◯

G. PATIENT SAFETY RATING

1. How would you rate your unit/work area on patient safety?	◯ Poor<br>◯ Fair<br>◯ Good<br>◯ Very good<br>◯ Excellent

H. HOSPITAL MANAGEMENT AND PATIENT SAFETY

	Strongly disagree	Disagree	Neither agree nor disagree	Agree	Strongly agree	Don't know

1. The actions by hospital management show that patient safety is a top priority	◯	◯	◯	◯	◯	◯
2. Hospital management provides adequate resources to improve patient safety	◯	◯	◯	◯	◯	◯
3. Hospital management only interested after an adverse event happens	◯	◯	◯	◯	◯	◯
4. When transferring a patient from one unit to another important information is often left	◯	◯	◯	◯	◯	◯
5. During shift changes/handover/ wardrounds important information is adequately exchanged	◯	◯	◯	◯	◯	◯

I. BARRIERS AND ENABLERS

1. What are the barriers to medical error reporting at KNH (Least at least two , that should be addressed) __________________________________

2. Mention an important enabler to medical error reporting, which ought to be reinforced within the institution of KNH __________________________________

3. List some improvement ideas for medical error reporting and patient safety in general __________________________________

END OF SURVEY
THANK YOU FOR PARTICIPATING IN THE SURVEY, THIS INFORMATION SHALL BE USED TO IMPROVE MEDICAL ERROR REPORTING AT KNH AND PATIENT SAFETY ____________________________
