# Supplementary material for: Medical Error Reporting among healthcare workers in a Kenyan tertiary level hospital: a knowledge, attitude, and practice study
Source: BMC Health Serv Res. 2025 Dec 17;26:102. doi: 10.1186/s12913-025-13886-0 (PMC12822039; doi:10.1186/s12913-025-13886-0)
Supplement: Supplementary file 2 — Supplementary Material 2 [file 12913_2025_13886_MOESM2_ESM.pdf]

CLINICAL ERRORS REPORTING TOOL REF: KNH/CORP/ME/F1/001

DISCLAIMER: The Hospital Management affirms that this is a blame free reporting tool. Any information provided is secure under Hospital policy and regulations and cannot be used against any staff for disciplinary action. The intent is to estimate the risk and evaluate patient safety for improved patient care.

(Please fill all areas provided)

1. Date of Report__________________ 2. Date of error__________________ 3. Time of Error______________

4. Patient Location at Time of Error (e.g. Ward No., Clinic No., other -specify): _________________________

5. Code Number (Generated) ____________________________

6. Age________ (years) 7. Gender: Male ☐ Female ☐ (please tick as applicable)

8 . Diagnosis (No abbreviations)

____

9. Types of Errors (please tick as applicable)

**Diagnostic**	**Treatment**	**Medication**	**Preventive**	**Others**
Error in diagnosis	Error in performance of surgery	Prescribing	Failure to provide prophylactic treatment	Failure of communication
Delay in diagnosis	Error in performance of a procedure	Wrong Time	Inadequate monitoring of treatment	Equipment failure
Failure to employ indicated tests	Error in performance of a test	Unauthorized Drug	Inadequate follow-up of treatment	Other system failure
Use of outmoded tests	Error in administering treatment	Error in the dose		
Failure to act on results of monitoring	Improper Dose	Wrong Drug Preparation		
Failure to act on results of testing	Error in the method of using a drug	Wrong Administration Techniques		
	Avoidable delay in treatment	Deteriorated Drugs		
	Avoidable delay in responding to an abnormal test			
	Inappropriate care/ Use of outmoded therapy			

10.Describe in brief how the error occurred

____
____
____
____
____
____
____

11. What was the outcome? ____

____
____
____
____
____

12. Possible causes and/or contributing factors (please tick most applicable)

a)	Communication problems	
b)	Inadequate information flow	
c)	Patient-related issues	
d)	Staff-related issues	
e)	Device/Equipment Failure	
f)	Lack of Device/Equipment	
g)	Inadequate policies and procedures	
h)	Others (specify)	

13. Reported by (Designation) ____________________ **Date** ____________________

14. Preventive /mitigation measures

____
____
____

Signed____________________**Designation**____________________**Date**____________________
